# The relationship of rice yield and quality with the utilization of temperature and light resources in regions at different altitudes

**DOI:** 10.3389/fpls.2026.1858767

**Published:** 2026-06-03

**Authors:** Ruijie Li, Yongheng Luo, Tao Lu, Ailing Wang, Jiarui Ni, Kun Chen, Yuanyuan Sun, Guanzhou Luo, Xiaojuan Yuan, Qin Liao, Yong Zhao, Zhonglin Wang, Zhiyuan Yang, Jun Ma, Yongjian Sun

**Affiliations:** 1 Crop Ecophysiology and Cultivation Key Laboratory of Sichuan Province, Sichuan Agricultural University, Chengdu, China; 2 Sichuan Climate Center, Sichuan Meteorological Bureau, Chengdu, China; 3 Xuanhan County Branch of Sichuan Agricultural Broadcasting and Television School, Xuanhan Bureau of Agriculture and Rural Affairs, Xuanhan, China; 4 Yibin Nanxi Agriculture, Animal Husbandry and Fishery Science and Technology Extension Station, Yibin Nanxi Bureau of Agriculture and Rural Affairs, Yibin, China

**Keywords:** altitude, light resources, quality, temperature resources, yield

## Abstract

**Introduction:**

Existing studies on the effects of altitude on rice yield and quality have drawn different conclusions. However, the underlying mechanisms of how altitude-induced differences in the utilization of temperature and light resources drive the differential responses of yield and quality formation remain unclear. In particular, the synergistic effects of the ecological adaptability of rice varieties, altitude-mediated distribution of light and temperature resources, and nitrogen regulation on rice yield and grain quality have not yet been clearly elucidated.

**Methods:**

A two-year field experiment was conducted in low-altitude (520.70 m) and high-altitude (1640.56 m) rice-growing regions using two rice varieties with differential altitude responsiveness of core agronomic traits, Meixiangzhan 2 and Yunjing 39. Under a nitrogen (N) application rate of 150 kg N ha−1, three N management strategies with basal: tiller: panicle fertilizer ratios of 5:3:2 (N1), 3:3:4 (N2), and 3:1:6 (N3) were implemented, with a no-N treatment (N0) as the control.

**Results:**

Altitude, variety, and N management significantly affected grain yield, rice quality, and the utilization of temperature and light resources. Compared with the low-altitude site, the rice growth period at high altitude was prolonged by 27.25-30.77 days, while effective panicles and seed-setting rates increased by 16.53%-27.37% and 2.71%-6.68%, respectively, contributing to a 4.10%-4.12% yield increase. Meixiangzhan 2 showed higher yields at low altitude (4.84%-8.12%) but significantly lower yields at high altitude (19.80%-25.13%) compared with Yunjing 39. High altitude improved grain quality by increasing head rice rate (5.10%-9.42%) and reducing chalkiness (2.87%-6.38%), although lower temperatures from the heading to maturity stage increased amylose content (13.71%-19.82%) and reduced taste value (2.29%-5.22%). Among the N treatments, N2 consistently improved both yield and rice quality of two varieties at different altitudes.

**Discussion:**

Optimizing temperature and light resource allocation during key growth stages-particularly increasing the effective accumulated temperature over the entire rice growth period, the diurnal temperature range from heading to maturity stage, the average daily sunshine hours, and the solar radiation—is critical for achieving high yield, high quality, and efficient resource utilization across altitudes.

## 1 Introduction

Rice is a fundamental food crop worldwide and a primary source of staple for billions (Chen et al., 2023a; Lu et al., 2022). With social progress and improved living standards, the demand for high-quality rice has been increasing. Thus, rice production has gradually shifted from blindly pursuing yield in the past to achieving synergistic quantity and quality enhancements (Song et al., 2025). While genetic characteristics, ecological conditions, and cultivation measures critically affect the yield and quality of different rice varieties (Jiang et al., 2015; Zhong et al., 2022), the breeding cycle of varieties with wide adaptability and breakthrough performance is long. Therefore, it is increasingly important to improve rice yield and quality through cultivation techniques and the utilization of ecological environment resources.

N fertilizer application is vital in rice production and among the key factors affecting rice growth, yield, and quality (Gu et al., 2017). The current N fertilizer application rate for single-season rice in China is 180 to 225 kg ha^−1^, far above the world average rate. Excessive N fertilizer application not only causes inefficient resource utilization and increases the economic burden of rice production, but also gives rise to a series of severe ecological and environmental risks (Dai and Zhang, 2020). The excessive N application restrict the synergistic improvement of yield and quality, hindering sustainable agriculture development (Jing et al., 2008; Zhou et al., 2022). Therefore, reducing N fertilizer application while improving rice yield and quality remains an important challenge in China’s rice production.

The various rice planting areas widely distributed across the globe represent complex and changeable ecological environments (Wu et al., 2021). Meanwhile, diurnal differences in light and temperature and the accumulated temperature are the main climatic factors affecting rice yield and quality (Feng et al., 2022), especially under different ecological conditions across altitudes. As altitude increases, the air density and temperature gradually decrease while the solar radiation intensity increases (Bennie et al., 2006; Liu et al., 2025). The resultant different climates affect rice growth and development differently, leading to diverse yield and quality outcomes. Relevant studies have shown that the main factors of rice yield differences are the number of effective panicles and the grain number per panicle. Weak solar radiation and low temperatures hinder spikelet differentiation, often decelerating grain filling and delaying maturity (Guo et al., 2024). These disadvantages directly affect the number of effective panicles and the grain number per panicle (Feng et al., 2022). Previous research also suggested that compared to high-altitude rice-growing regions with greater light resources, the number of effective panicles in low-altitude rice-growing regions with lower light resources is significantly reduced, which explains the significantly lower yield (Zhong et al., 2022). However, some studies have shown that decreased rice yield and yield components with the increase of altitude and identified the main factors explaining yield differences across altitudes as the number of effective panicles and the grain number per panicle (Zheng et al., 2020a). The effects of ecological conditions at various altitudes on rice quality have been extensively studied, but the findings diverged significantly. Previous studies reported that high temperature during the grain-filling stage could increase the gelatinization temperature and gelatinization enthalpy, change the crystalline structure and the component of starch, and consequently result in poor eating and cooking quality in rice (Kong et al., 2015; Yao et al., 2020). Low temperature during the grain filling stage increase the chalkiness rate and chalkiness of rice, decrease the starch and amylopectin content, and consequently lead to a decline in appearance and nutritional quality (Yang et al., 2024). Temperature difference affects grain filling accumulation and significantly impacts rice processing quality (Song et al., 2013).

The southwest area is one of the seven physical geographical subregions in China and one of the main rice planting areas (Fu et al., 2022). In 2019, the area under rice cultivation in Sichuan and Chongqing was 25,251,000 ha, with a yield of 19,568,000 tons, accounting for 8.55% and 9.34%, respectively, of total rice cultivation area and production nationwide (Yang et al., 2022). Thus, ensuring rice yield stability and increment in this region is crucial for China’s national food security. However, Southwest China features diverse landforms, such as plains, hills, and mountains, and a vast altitude range of 76 to 2710 m. Its three-dimensional climate characteristics form a significant regional light and heat resource gradient (Sun et al., 2015). In contrast to the typical “high temperature and high solar radiation” conditions prevailing in the middle and lower reaches of the Yangtze River regions, the southwest area is characterized by frequent cloud cover, high relative humidity, and relatively low solar radiation (Fu et al., 2022). These complex climatic conditions have led to a unique pattern of light-temperature imbalance. At low altitudes, rice plants suffer combined stress of high temperature and weak solar radiation during the grain-filling period; by contrast, rice cultivation at high altitudes is mainly restricted by insufficient accumulated temperature (Wu et al., 2020).

The interaction between variety and altitude has significant effects on rice yield formation and grain quality performance. Studies conducted by researchers under different altitude conditions in Southwest China have shown that super hybrid rice varieties respond markedly differently to altitudinal gradients: some varieties exhibit stronger adaptability under high-altitude conditions characterized by low temperature and high radiation, while others display more pronounced yield advantages under low-altitude conditions with high temperature and low radiation (Jiang et al., 2025). The regulatory effects of the interaction between N fertilizer and variety on rice yield and quality formation have also been extensively studied. It has been found that increasing the proportion of N applied at the late growth stage can significantly improve the eating quality of high-quality indica rice; however, the response to an increased proportion of panicle N fertilizer is genotype-specific, and the eating quality of some varieties may actually decline due to excessive accumulation of protein content (Xiong et al., 2024). For a long time, due to blind interregional variety introduction and uniform N fertilizer management in the southwestern rice region, there has been a prominent mismatch among the ecological adaptability of rice varieties, the ecological environment, and field management practices, resulting in significant differences in rice yield and quality across Southwest China. This interaction effect among varieties, environment, and N management has often been overlooked in studies focusing on plain rice-growing areas, yet it represents a critical bottleneck restricting further yield improvement in rice production in the southwestern rice region.

Previous studies have yielded diverse conclusions concerning the effects of altitude on rice yield and quality and identified inconsistent factors to explain yield changes. Few conducted dynamic analysis of the distribution of temperature and light resources throughout the rice growth period, and research on the complex mountain environment in Southwest China is scarce. Although previous studies have explored the single-factor effects as well as the interactions of variety with N management and altitude with variety on rice yield and grain quality, research focusing solely on the altitude–variety interaction cannot resolve how to regulate and optimize rice traits through cultivation practices. Meanwhile, studies examining the variety with N management interaction have largely neglected the modulating effects of altitude-driven gradients in light and temperature resources on variety performance and N fertilizer efficacy. The ecological adaptability of rice varieties, the altitude-mediated distribution of photo-thermal resources, and N regulatory effects are interconnected; neither single-factor analyses nor two-factor interaction studies are sufficient to address the core production challenges confronting the southwestern rice region. In the context of promoting N reduction and efficiency improvement and ensuring food quality and safety, achieving simultaneous optimization of rice yield and quality across altitudes through precise N fertilizer management still requires a systematic theoretical basis and technical support. Building upon previous studies (Yang et al., 2022; Sun et al., 2015; Wu et al., 2020), this research considers the complex ecological environment in the Southwest China rice-growing region, adopts the stable and efficient reduction of N application rate (150 kg ha^−1^) and different N fertilizer operations to explore the differences in yield and quality of different rice varieties at different altitudes, aiming to isolate the main limiting factors for yield and quality improvements. The findings are of great significance to developing N fertilizer control technologies to enhance quality and efficiency and are expected to contribute to the balance among high-yield, high-quality, and high-efficiency in rice cultivation across Southwest China.

## 2 Materials and methods

### 2.1 Test site and materials

**Figure 1:**
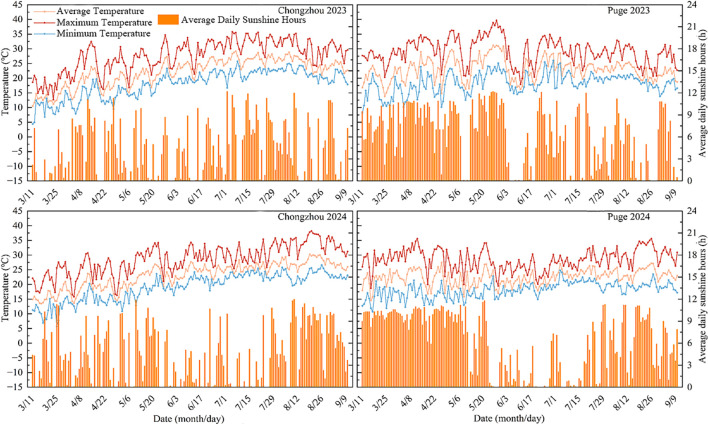
Meteorological data for rice growing seasons at different altitudes.

The experiment was conducted in 2023 and 2024 in the modern agricultural research and development base of Sichuan Agricultural University in Chongzhou City, Sichuan Province (a typical test base of rice production in western Sichuan Plain, 30°33′N, 103°38′E, altitude 520.70 m) and Dapingzi Village, Qiaowo Town, Puge County (a typical test base in southwest plateau, 27°27′N, 102°29′E, altitude 1640.56 m). The soil texture at the two ecological sites was sandy loam, and the nutrient content of the plough layer (0 to 20 cm deep) is shown in **Table 1**. The agrometeorological data in the rice-growing seasons are shown in **Figure 1**. Following a multi-criteria selection framework based on rice subspecies classification, normal maturity capability under high-altitude environmental conditions, and differential altitude responsiveness of core agronomic traits, two conventional rice cultivars exhibiting similar full growth cycle durations were chosen as experimental materials: conventional *indica* rice Meixiangzhan 2 (provided by Xichang College) and conventional *japonica* rice Yunjing 39 (provided by Yunnan Academy of Agricultural Sciences).

**Table 1:** Soil physical and chemical properties of the soil at different altitudes.

Year	Organic matter (g kg^-1^)	Total N (g kg^-1^)	Available N (mg kg^-1^)	Total P (g kg^-1^)	Available P (mg kg^-1^)	Total K (g kg^-1^)	Available K (mg kg^-1^)
Low altitude							
2023	27.13	1.71	80.63	1.28	15.54	3.36	97.43
2024	28.29	1.79	85.46	1.14	15.29	3.48	94.82
High altitude							
2023	21.75	1.81	75.28	1.04	10.82	3.82	109.86
2024	22.52	1.93	81.74	0.93	10.56	3.77	112.31

### 2.2 Experimental design

The field trial at the two ecological sites was arranged in a three-factor split-plot design. The main plot factor was ecological site, rice cultivar, and the subplot- factor consisted of N fertilization regimes. Four N treatments were established, comprising three distinct N management practices (provided by the Shanghai Research Institute of Chemical Industry), with basal: tiller: panicle N fertilizer ratios of 50:30:20, 30:30:40, and 30:10:60, respectively. A no-N treatment was included as the control. These treatments were denoted as N_1_, N_2_, N_3_, and N_0_, respectively. Each treatment was replicated three times, with an individual plot area of 21.6 m^2^ (5.4 m in length and 4 m in width), resulting in a total of 24 experimental plots at each ecological site. All plots were isolated by 40 cm-high and 30 cm-wide ridges lined with plastic film, and a 1.0-m-wide protective buffer row was established around each plot to eliminate lateral nutrient and water interference between adjacent plots.

For a uniform total N application rate of 150 kg ha^-1^, the ratio of basal fertilizer to tiller fertilizer in the combined basal-tillering N input was set at 5:5. N fertilizer (urea, 46.0% N) was applied as basal dressing one day before transplanting; tiller fertilizer was supplied seven days after transplanting; panicle fertilizer was equally split and applied at the emergence of the 4th top leaf and the 2nd top leaf, respectively. Phosphorus fertilizer (superphosphate, 12% P_2_O_5_) was applied once at a rate of 75 kg P_2_O_5_ ha^-1^ as basal fertilizer one day prior to transplanting. Potassium fertilizer (potassium chloride, 60% K_2_O) was applied at 150 kg K_2_O ha^-1^, equally split between basal fertilization and jointing stage fertilization, which wereximplemented one day before transplanting and at the emergence of the 4th top leaf, respectively. The application rates and methods of phosphorus and potassium fertilizers in the no-N treatment remained consistent with those adopted in the N-fertilized treatments.

At both ecological sites, a plastic film arch shed was used to maintain temperature and humidity for the seedlings, and seedlings at the four-leaf stage were manually transplanted. The row spacing was 30 cm × 18 cm, with two seedlings per hill. A continuous shallow water layer was maintained throughout the tillering stage. Field drainage and mild intermittent soil drying were initiated at one leaf age prior to the critical stage of effective tillering, when the population stem and tiller number reached approximately 80% of the targeted panicle number. Wet irrigation was adopted from the jointing stage to physiological maturity, and field water was withheld 5–7 days prior to harvesting. Pest, disease, and weed control were conducted following local standardized large-scale rice production practices.

### 2.3 Determination items and methods

#### 2.3.1 Meteorological data

The meteorological data throughout the rice growth period at the two ecological sites were acquired from the Sichuan Provincial Climate Center. The specific data included average daily temperature, daily maximum temperature, daily minimum temperature, sunshine hours, and daily average radiation.

#### 2.3.2 Photosynthetic productivity

At the jointing, heading, and maturity stages, 5 hills of representative plants were collected from each plot (with the number of tillers consistent with the average of the plot) and separated into stem sheath, leaves, and panicles. After being fixed at 105°C for 30 min, the samples were dried at 80°C to constant weight for subsequent analyses.

#### 2.3.3 Seed test and yield test

At the mature stage, 5 hills of rice consistent with the average number of tillers in the plot were harvested from each plot. The number of empty grains, the number of filled grains, and the thousand kernel weight were investigated in the laboratory, and the seed setting rate was calculated. The edge row of each plot was removed, and rice was harvested from the remaining 12.0 m^2^. The yield was calculated based on the standard water content (13.5% for indica rice and 14.5% for japonica rice).

#### 2.3.4 Rice quality

After harvest, 1.0 kg of rice was naturally dried in the shade for 3 months, and the brown rice rate, head rice rate, and chalkiness traits were determined by the conventional physical weighing methods (Wei et al., 2024; Shi et al., 2021). Then, 30.0 g of rice was cooked and cooled at a 1:1.3 rice-to-water ratio for 2 h, and the taste value was determined by the STA1 B rice taste meter (Satake Company, Japan). The protein content was measured by multiplying the N content of brown rice determined by the Kjeldahl method by 5.95. Amylose content was determined by the colorimetric method (Bhat and Riar, 2016).

#### 2.3.5 Parametric computation

The relevant indicators were calculated as follows (Sun et al., 2015; Wu et al., 2020; Huang et al., 2022; Wu et al., 2022a):

1. Dry matter accumulation (kg ha^−1^) = (stem sheath + leaf + panicle) dry weight.
2. Dry matter transport amount of stem-sheath and leaf (kg ha^−1^) = stem-sheath and leaf dry weight at heading stage − stem-sheath and leaf dry weight at maturity stage.
3. Dry matter transport rate of stem-sheath and leaf (%) = stem-sheath and leaf dry matter translocation amount/stem-sheath and leaf dry weight at the heading stage×100.
4. Dry matter contribution rate of stem-sheath and leaf (%) = translocation amount of stem-sheath and leaf dry matter/dry weight of panicle at mature stage×100.
5. Production efficiency of effective accumulated temperature (kg ha^−1^ °C^−1^) = yield/effective accumulated temperature during the whole growth period.
6. Solar radiation production efficiency (g MJ^−1^) = yield/solar radiation accumulation during the whole growth period.
7. Effective accumulated temperature (°C) =∑[average daily temperature−10°C]×growth days.
8. Diurnal temperature range (°C) = daily maximum temperature − daily minimum temperature.

### 2.4 Data processing and analysis

Microsoft Excel 2016 was used for data processing. Pearson correlation analysis was performed to examine relationships between variables, and the least significant difference (LSD) test with a probability level of 0.05 was used to determine the significance of differences between treatments. IBM SPSS Statistics 27 was used for variance analysis, paired t-test, principal component analysis, and membership function analysis. Origin 2024 was employed to plot the Figure. The equations related to identifying the optimal treatment are as follows (Song et al., 2024):

The subordinate function values were calculated as follows:


$$\text{μ}\left({\text{X}}_{\text{i}}\right)=\left({\text{X}}_{\text{i}}-{\text{X}}_{\text{min}}\right)/\left({\text{X}}_{\text{max}}-{\text{X}}_{\text{min}}\right),\;i=1,2,3,\cdots ,\text{n}$$

where X_i_ represents the *i-*th comprehensive index, X_min_ represents the minimum value of the *i-*th comprehensive index; and X_max_ denotes the maximum value of the *i*-th comprehensive index.

The weight was calculated as follows:


$${\text{W}}_{\text{i}}={\text{P}}_{\text{i}}/\sum_{\text{i}=1}^{\text{n}}{\text{P}}_{\text{i}}$$

where W_i_ represents the weight of the *i*-th principal component, and P_i_ is the contribution rate of the *i*-th comprehensive index.

The comprehensive evaluation score was calculated as follows:


$$\text{D}=\sum_{\text{i}=1}^{\text{n}}\left[\mu ({\text{X}}_{\text{i}})\times {\text{W}}_{\text{i}}\right]$$

where D value represents the comprehensive evaluation score of each treatment affecting rice yield.

## 3 Results

### 3.1 Growth period, yield, and yield composition

Different altitudes, varieties, and N application treatments significantly affected the growth period (**Table 2**). The growth periods of Meixiangzhan 2 and Yunjing 39 at the high altitude increased significantly by 27.25 to 30.77 days. According to **Table 3**, different altitudes significantly impacted the number of effective panicles, grain number per panicle, and seed setting rate in 2023 and 2024. Compared to the low altitude, the grain number per panicle at the high altitude was significantly reduced by 17.19% to 20.57%, whereas the effective panicle number and seed setting rate were significantly increased by 16.53% to 27.37% and 2.71% to 6.68%, respectively. Those increments led to yield increases by 4.10% to 4.12% at the high altitude.

**Table 2:** Main growth period of rice under different altitude locations.

Altitude	Variety	N management	Sowing stage (Month/Day)		Transplanting stage (Month/Day)		Jointing stage (Month/Day)		Heading stage (Month/Day)			Maturity stage (Month/Day)		Whole growth period (Day)	
			2023	2024	2023	2024	2023	2024	2023	2024		2023	2024	2023	2024
Low altitude	MXZ 2	N_0_	4/8	4/5	5/9	5/5	6/21	6/18	7/18		7/16	8/19	8/16	133	133
		N_1_	4/8	4/5	5/9	5/5	6/22	6/19	7/18		7/16	8/21	8/18	135	135
		N_2_	4/8	4/5	5/9	5/5	6/24	6/21	7/18		7/17	8/22	8/19	136	136
		N_3_	4/8	4/5	5/9	5/5	6/23	6/20	7/19		7/18	8/24	8/21	138	138
	YJ 39	N_0_	4/8	4/5	5/9	5/5	6/18	6/15	7/16		7/11	8/18	8/15	132	132
		N_1_	4/8	4/5	5/9	5/5	6/19	6/16	7/15		7/11	8/20	8/17	134	134
		N_2_	4/8	4/5	5/9	5/5	6/21	6/18	7/16		7/12	8/21	8/18	135	135
		N_3_	4/8	4/5	5/9	5/5	6/20	6/17	7/15		7/11	8/22	8/19	136	136
High altitude	MXZ 2	N_0_	3/23	3/15	4/30	4/26	6/26	6/24	8/1		7/28	9/8	9/5	169	174
		N_1_	3/23	3/15	4/30	4/26	6/27	6/25	8/2		7/28	9/11	9/8	172	177
		N_2_	3/23	3/15	4/30	4/26	6/28	6/26	8/3		8/1	9/12	9/9	173	178
		N_3_	3/23	3/15	4/30	4/26	6/26	6/24	8/4		7/29	9/14	9/10	175	179
	YJ 39	N_0_	3/23	3/15	4/30	4/26	6/23	6/22	7/26		7/26	9/8	9/4	169	173
		N_1_	3/23	3/15	4/30	4/26	6/24	6/23	7/28		7/26	9/9	9/6	170	175
		N_2_	3/23	3/15	4/30	4/26	6/25	6/24	7/29		7/27	9/11	9/8	172	177
		N_3_	3/23	3/15	4/30	4/26	6/23	6/22	7/28		7/26	9/12	9/9	173	178

MXZ 2: Meixiangzhan 2; YJ 39: Yunjing 39; N_0_ represents a blank control with no N fertilizer application, N_1_, N_2_, and N_3_ represent three N management strategies with a total N application rate of 150 kg ha^-1^ and basal:tiller:panicle fertilizer ratios of 5:3:2, 3:3:4, and 3:1:6, respectively.

**Table 3:** Effects of variety and N management on grain yield and its components under different altitude locations.

Year	Altitude	Variety	N management	Effective panicles (×10^4^ ha^-1^)	Grain number per panicle	Total spikelets (×10^6^ ha^-1^)	Seed setting rate (%)	Thousand kernel weight (g)	Grain yield (kg ha^-1^)
2023	Low altitude	MXZ 2	N_0_	206.04e	181.02d	372.97d	87.59a	20.75d	6411.65f
			N_1_	264.56a	183.60c	485.74b	83.66b	20.73d	8095.68b
			N_2_	263.20ab	191.72a	504.59a	83.58b	21.14c	8471.08a
			N_3_	260.50b	186.46b	485.73b	81.62c	20.85d	7893.70c
			**Average**	**248.58**	**185.70**	**462.26**	**84.11**	**20.87**	**7718.03**
		YJ 39	N_0_	191.35f	151.95g	290.76f	84.58b	27.34b	6294.93f
			N_1_	242.04c	154.56f	374.10d	81.40c	27.30b	7723.24d
			N_2_	238.70c	162.87e	388.75c	81.26c	27.84a	8350.28a
			N_3_	221.77d	156.64f	347.41e	79.42d	27.66a	7080.62e
			**Average**	**223.47**	**156.51**	**350.26**	**81.66**	**27.54**	**7362.27**
	High altitude	MXZ 2	N_0_	261.61f	116.92g	305.86g	89.77a	20.91d	5254.78h
			N_1_	321.07bc	131.59f	422.49e	86.85bc	20.11f	6806.63g
			N_2_	334.66a	134.89e	451.41b	86.22c	21.12d	7833.42d
			N_3_	322.65b	135.22e	436.28d	84.17d	20.63e	6990.72f
			**Average**	**309.99**	**129.65**	**404.01**	**86.75**	**20.69**	**6721.39**
		YJ 39	N_0_	235.11g	136.94d	321.96f	87.06b	27.64ab	7263.25e
			N_1_	308.33d	140.63c	433.63d	83.83d	27.35c	9443.05c
			N_2_	318.11c	144.42b	459.40a	82.97e	27.82a	10076.68a
			N_3_	303.51e	146.71a	445.27c	80.24f	27.48bc	9327.45b
			**Average**	**291.27**	**142.17**	**415.06**	**83.33**	**27.57**	**8977.61**
		*F* value	A	11572.72**	10633.67**	7.73**	108.76**	2.80	202.63**
			V	1331.81**	596.83**	1831.06**	172.60**	26169.58**	1723.94**
			N	2535.06**	227.00**	2387.21**	127.04**	35.10**	1827.35**
			A×V	28.23**	3734.49**	2720.76**	3.24	6.47*	3211.12**
			A×N	93.49**	36.62**	56.60**	1.14	7.46**	53.77**
			V×N	16.46**	14.09**	30.96**	0.17	1.84	11.84**
			A×V×N	30.00**	14.09**	17.99**	0.93	3.53*	27.76**
2024	Low altitude	MXZ 2	N_0_	217.19	168.13c	365.14de	89.12a	21.08cd	6562.82e
			N_1_	274.57a	173.57b	476.57b	85.65b	20.93d	8261.35b
			N_2_	270.98b	177.93a	482.14a	84.81bc	21.38c	8494.74a
			N_3_	267.52c	176.21ab	471.42b	83.31d	20.71d	7929.14c
			**Average**	**257.57**	**173.96**	**448.82**	**85.72**	**21.02**	**7812.01**
		YJ 39	N_0_	192.54h	146.26f	281.52f	84.02cd	27.53b	6110.19f
			N_1_	245.13d	149.39e	366.19d	79.02e	27.56b	7436.85d
			N_2_	241.06e	157.64d	380.01c	78.29ef	28.34a	8024.13c
			N_3_	229.24f	157.16d	360.25e	77.13f	27.72b	7329.36d
			**Average**	**226.99**	**152.61**	**346.99**	**79.62**	**27.79**	**7225.13**
	High altitude	MXZ 2	N_0_	240.96e	120.33e	289.94d	90.24a	21.08de	5347.53h
			N_1_	301.17c	133.52d	402.12c	89.82ab	20.71f	7210.95f
			N_2_	316.20a	135.52bc	428.48a	89.05b	21.19d	7814.47d
			N_3_	310.22b	136.06b	422.08ab	87.16c	20.90ef	7499.27e
			**Average**	**292.14**	**131.36**	**385.65**	**89.07**	**20.97**	**6968.06**
		YJ 39	N_0_	217.72f	133.06d	289.67d	89.03b	27.87ab	6818.97g
			N_1_	286.67d	137.07b	392.95c	87.96c	27.12c	8846.81c
			N_2_	295.93c	142.08a	420.557ab	87.66c	27.94a	9965.04a
			N_3_	289.85d	144.08a	417.62b	84.59d	27.64b	9120.80b
			**Average**	**272.54**	**139.07**	**380.20**	**87.31**	**27.64**	**8687.91**
		*F* value	A	2241.41**	3775.50**	92.84**	870.37**	4.36*	137.44**
			V	878.73**	222.52**	1190.40**	441.45**	19360.85**	460.71**
			N	1279.65**	139.77**	1315.89**	124.70**	31.19**	1489.41**
			A×V	42.12**	1011.64**	960.50**	135.19**	1.01	1909.78**
			A×N	59.69**	4.96**	29.45**	20.91**	6.84**	68.71**
			V×N	3.37*	7.57**	6.68**	2.15	3.27*	12.80**
			A×V×N	4.57**	4.12*	2.62	0.93	2.24	8.26**

MXZ 2, Meixiangzhan 2; YJ 39, Yunjing 39; N_0_ represents a blank control with no N fertilizer application, N_1_, N_2_, and N_3_ represent three N management strategies with a total N application rate of 150 kg ha^-1^ and basal:tiller:panicle fertilizer ratios of 5:3:2, 3:3:4, and 3:1:6, respectively. Different letters after the same column data indicate significant differences at the 5% level between treatments in the same location. A, Altitude; V ,variety; N ,N management; A×V ,altitude and variety management interaction; A×N, altitude and N management interaction; V×N ,variety and N management interaction; A×V×N, altitude, variety and N management interaction. *, ** indicate significant differences at the 0.05 and 0.01 levels, respectively.

Bold values represent the average values of the same indicator for the same variety under four nitrogen fertilizer treatments. The lowercase letters in the table are used for statistical significance marking. Different letters indicate significant differences among treatments at the same ecological site at the 5% level, while the same letter indicates no significant difference.

Varieties and N fertilizer management significantly influenced rice yield and yield components at the two altitudes. The interaction between varieties and N fertilizer management showed significant effects on effective panicles, grain number per panicle, total spikelets, and yield. The interactive effects of altitude, variety and N management on effective panicle, grain number per panicle, and grain are significant. The yield of each variety varied across altitudes. The yield of Meixiangzhan 2 at the low altitude was 4.84% to 8.12% higher than that of Yunjing 39, and that at the high altitude was significantly lower than Yunjing 39 by 19.80% to 25.13%. Yield component analysis showed that the grain number per panicle of Meixiangzhan 2 at the low altitude decreased significantly by 24.49% to 30.18% compared to the high altitude, resulting in a significant yield decrease of 12.11% to 14.82%. In contrast, although the grain number per panicle of Yunjing 39 at the low altitude decreased by 8.87% to 9.16% compared to the high altitude, the effective panicles and seed setting rate increased by 20.27% to 30.34% and 2.28% to 9.66%, respectively, leading to yield increments by 20.25% to 21.94%. The yield of all varieties at different altitudes increased first and then decreased with the increase in the proportion of postponed N fertilizer. The yield of the N_2_ treatment was the highest, which was 29.44% to 49.07%, 2.83% to 15.09%, and 4.20% to 17.93% higher than that of N_0_, N_1_, and N_3_ treatments, respectively.

### 3.2 Photosynthetic production and translocation

Altitude significantly affected the total dry matter accumulation and the dry matter accumulation from the sowing to the jointing stage and from jointing to the heading stage (**Table 4**). At all altitudes, the dry matter accumulation of Yunjing 39 exceeded that of Meixiangzhan 2 from sowing to jointing and from heading to maturity, but was significantly exceeded by Meixiangzhan 2 from jointing to heading. The total dry matter accumulation of each variety differed at different altitudes. The total dry matter accumulation of Meixiangzhan 2 at the low altitude was 2.55% to 4.90% higher than that of Yunjing 39, while that at the high altitude was significantly lower by 10.21% to 13.70%. Regarding the effects of N fertilizer management on dry matter accumulation at each rice growth stage, the N_1_ treatment led to the highest dry matter accumulation across varieties and altitudes from sowing to the jointing stage. After jointing, i.e., from jointing to heading and from heading to maturity, the dry matter accumulation at the low altitude increased first and then decreased with the increase of the proportion of postponed N fertilizer application, peaking under the N_2_ treatment. At the high altitude, the material accumulation of the two varieties from jointing to heading and from heading to maturity was the highest under the N_3_ treatment.

**Table 4:** Effects of variety and N management on dry matter accumulation of rice under different altitude locations.

Year	Altitude	Variety	N management	Dry matter accumulation (kg ha^-1^)			Total dry matter accumulation (kg ha^-1^)
				Sowing-jointing stage	Jointing-heading stage	Heading-maturity stage	
2023	Low altitude	MXZ 2	N_0_	2592.64f	5375.02e	4003.63e	11971.29f
			N_1_	3881.68b	6614.76b	5429.41c	15925.84ab
			N_2_	3503.87d	7013.50a	5483.89c	16001.26a
			N_3_	3259.68e	6279.07c	5268.86d	14807.60d
			**Average**	**3309.47**	**6320.59**	**5046.45**	**14676.50**
		YJ 39	N_0_	3220.07e	4185.55g	4036.83e	11442.44g
			N_1_	4203.63a	5374.71f	5811.54b	15389.88c
			N_2_	3878.67b	5796.94d	6149.85a	15825.45b
			N_3_	3639.13c	5241.67f	5708.53b	14589.34e
			**Average**	**3735.37**	**5149.72**	**5426.69**	**14311.78**
	High altitude	MXZ 2	N_0_	2297.55g	4636.53d	2855.24g	9789.31g
			N_1_	3673.56c	5209.89c	4401.27f	13284.72e
			N_2_	3441.75d	6122.28a	4773.54e	14337.56c
			N_3_	2861.32e	6269.25a	4930.26d	14060.83d
			**Average**	**3068.55**	**5559.49**	**4240.08**	**12868.10**
		YJ 39	N_0_	2649.16f	4101.88e	4667.59e	11418.63f
			N_1_	4219.78a	4829.15d	6409.42c	15458.35b
			N_2_	4031.71b	5655.82bc	6740.59b	16428.12a
			N_3_	3576.72cd	5845.16b	6913.46a	16335.34a
			**Average**	**3619.34**	**5108.00**	**6182.77**	**14910.11**
		*F* value	A	52.24**	138.45**	0.85	762.26**
			V	391.11**	565.40**	1804.32**	1464.54**
			N	522.84**	411.67**	1074.21**	8738.68**
			A×V	6.40*	111.17**	816.31**	3015.38**
			A×N	17.01**	59.44**	33.66**	336.26**
			V×N	0.88	0.72	9.43**	23.18**
			A×V×N	7.62**	0.64	3.34*	7.84**
2024	Low altitude	MXZ 2	N_0_	2639.63f	5522.70e	4158.72f	12321.05f
			N_1_	4012.75a	6416.03b	5625.94c	16054.72a
			N_2_	3721.50c	6727.82a	5712.17bc	16161.49a
			N_3_	3476.38d	6227.55c	5300.33e	15004.26d
			**Average**	**3462.57**	**6223.53**	**5199.29**	**14885.38**
		YJ 39	N_0_	2873.67e	4458.88f	4079.84f	11412.39g
			N_1_	4113.5a	5503.31e	5772.09ab	15388.90c
			N_2_	3887.84b	5771.39d	5856.28a	15515.52b
			N_3_	3587.95d	5393.13e	5463.91d	14445e
			**Average**	**3615.74**	**5281.68**	**5293.03**	**14190.45**
	High altitude	MXZ 2	N_0_	2103.70g	4652.17c	3220.22h	9976.09g
			N_1_	3483.78c	5514.18b	4387.9g	13385.86e
			N_2_	3332.35d	6243.10a	4758.22e	14333.67c
			N_3_	2896.90e	6329.14a	4991.01d	14217.06d
			**Average**	**2954.18**	**5684.65**	**4339.34**	**12978.17**
		YJ 39	N_0_	2437.71f	4248.04d	4503.00f	11188.75f
			N_1_	3981.42a	4745.36c	6101.01c	14827.78b
			N_2_	3798.96b	5566.98b	6567.99b	15933.93a
			N_3_	3469.54c	5660.26b	6771.90a	15901.70a
			**Average**	**3421.91**	**5055.16**	**5985.97**	**14463.04**
		*F* value	A	233.361**	174.20**	12.72**	2116.40**
			V	182.451**	734.18**	1381.78**	494.25**
			N	701.201**	432.15**	1214.98**	12120.23**
			A×V	46.831**	29.01**	1100.11**	3763.56**
			A×N	4.921**	54.31**	77.42**	579.34**
			V×N	0.29	0.77	14.80**	24.81**
			A×V×N	2.94*	4.25*	2.04	1.13

MXZ 2, Meixiangzhan 2; YJ 39, Yunjing 39; N_0_ represents a blank control with no N fertilizer application, N_1_, N_2_, and N_3_ represent three N management strategies with a total N application rate of 150 kg ha^-1^ and basal:tiller:panicle fertilizer ratios of 5:3:2, 3:3:4, and 3:1:6, respectively. Different letters after the same column data indicate significant differences at the 5% level between treatments in the same location. A, Altitude; V, variety; N, N management; A×V, altitude and variety management interaction; A×N, altitude and N management interaction; V×N, variety and N management interaction; A×V×N, altitude, variety and N management interaction. *, ** indicate significant differences at the 0.05 and 0.01 levels, respectively.

Bold values represent the average values of the same indicator for the same variety under four nitrogen fertilizer treatments. The lowercase letters in the table are used for statistical significance marking. Different letters indicate significant differences among treatments at the same ecological site at the 5% level, while the same letter indicates no significant difference.

**Figure 2:**
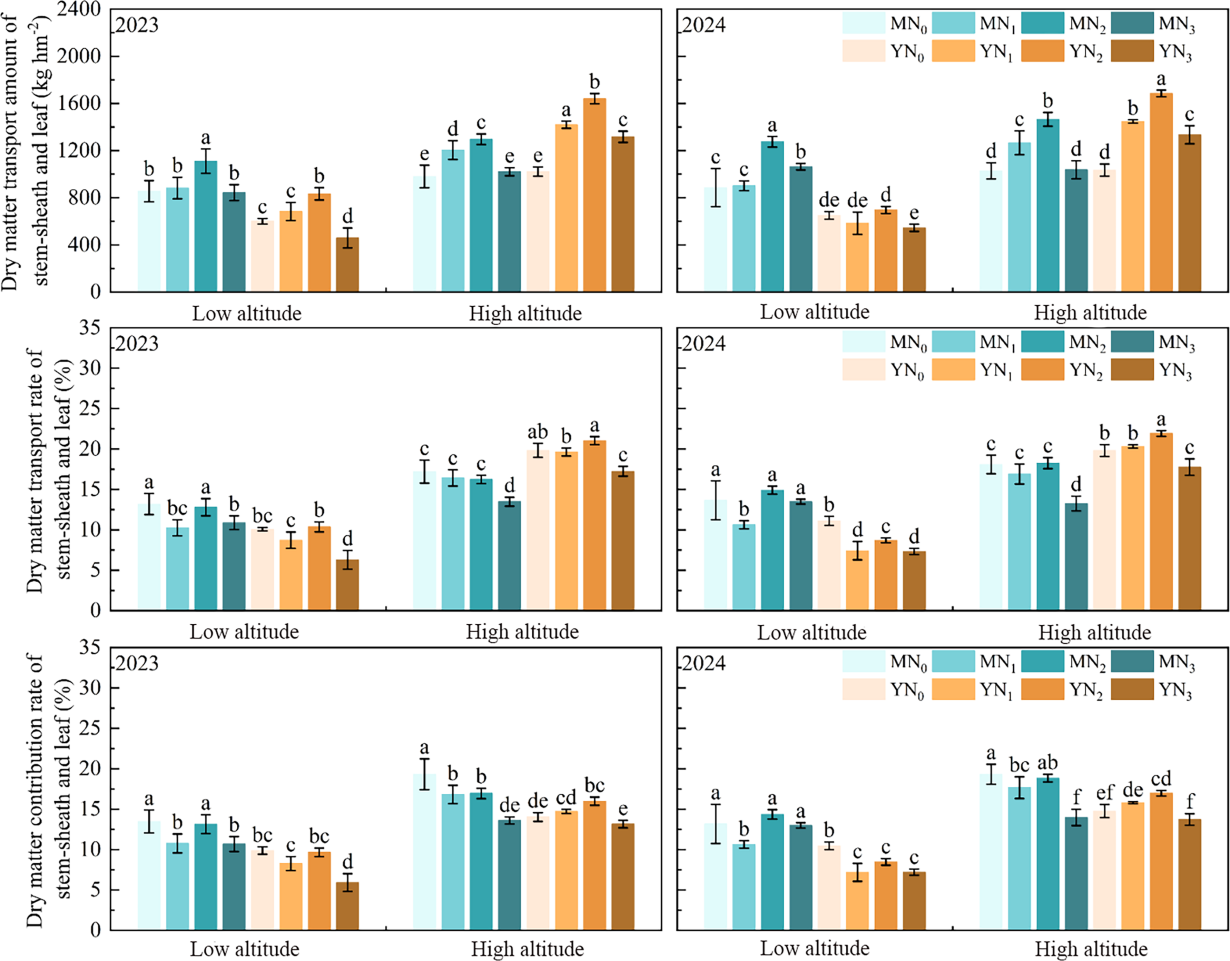
Effects of N management on material transport in rice at different altitude locations. MN_0_, MN_1_, MN_2_, and MN_3_ represent the treatment combinations of the conventional *indica* rice variety Meixiangzhan 2 under four N treatments (N_0_: no N application; N_1_: basal: tillering: panicle fertilizer ratios of 5:3:2; N_2_: basal: tillering: panicle fertilizer ratios of 3:3:4; N_3_: basal: tillering: panicle fertilizer ratios of 3:1:6), respectively. YN_0_, YN_1_, YN_2_, and YN_3_ represent the treatment combinations of the conventional *japonica* rice variety Yunjing 39 under four N treatments (N_0_: no N application; N_1_: basal: tillering: panicle fertilizer ratios of 5:3:2; N_2_: basal: tillering: panicle fertilizer ratios of 3:3:4; N_3_: basal: tillering: panicle fertilizer ratios of 3:1:6), respectively. The error bars in the Figure represent the standard deviation. Different lowercase letters indicate significant differences between different crop rotation systems at the 0.05 level.

**Figure 2** shows significant differences in the stem-sheath (leaf) dry matter translocation amount and rate at different altitudes. The stem-sheath and leaf dry matter translocation amount, translocation rate, and contribution rate at the high altitude were significantly higher than those at the low altitude, with an average increase of 56.03% to 57.98%, 3.76% to 11.82%, and 5.27% to 9.09% in the two years, respectively. The stem-sheath and leaf dry matter translocation amount, translocation rate, and contribution rate across varieties and altitudes varied. The average stem-sheath and leaf translocation amount, translocation rate, and contribution rate of Meixiangzhan 2 at the low altitude were significantly higher than those of Yunjing 39 in the two years, while those indicators at the high altitude were significantly lower than those of Yunjing 39 by 13.92% to 15.23%. At different altitudes, the stem-sheath and leaf translocation amount, translocation rate, and contribution rate of each variety were the highest under the N_2_ treatment. However, further increasing the proportion of postponed N fertilizer application (N_3_ treatment) was not conducive to the material transport from various vegetative organs in the fruiting stage.

### 3.3 Rice quality

As shown in MXZ 2: Meixiangzhan 2; YJ 39: Yunjing 39; N_0_ represents a blank control with no N fertilizer application, N_1_, N_2_, and N_3_ represent three N management strategies with a total N application rate of 150 kg ha^-1^ and basal:tiller:panicle fertilizer ratios of 5:3:2, 3:3:4, and 3:1:6, respectively. Different letters after the same column data indicate significant differences at the 5% level between treatments in the same location. A: Altitude; V: variety; N: N management; A×V: altitude and variety management interaction; A×N: altitude and N management interaction; V×N: variety and N management interaction; A×V×N: altitude, variety and N management interaction. *, ** indicate significant differences at the 0.05 and 0.01 levels, respectively.

**Table 5**, different altitudes significantly affect rice processing and appearance indicators. Compared with the low-altitude region, the high altitude significantly increased brown rice rate, milled rice rate, and head rice rate by 1.96% to 2.50%, 2.37% to 3.73%, and 5.10% to 9.42%, respectively. Meanwhile, the chalky grain rate, chalkiness, and length-width ratio were significantly reduced by 9.33% to 13.47%, 2.87% to 6.38%, and 3.96% to 4.89%, respectively. Variety and N application significantly affected all rice processing and appearance indicators, except the chalkiness ratio in 2024. The interactive effects of altitude, variety and N management on brown rice rate, chalky grain rate, and chalkiness ratio are significant. Yunjing 39 exhibited significantly better brown rice rate, milled rice rate, and head rice rate than Meixiangzhan 2 at different altitudes. However, Meixiangzhan 2 showed significantly lower chalky grain rate and chalkiness degree than Yunjing 39, and its length-width ratio was higher. Across the N treatments, the N_2_ treatment at the low altitude was beneficial to improve the processing quality of each variety, while the N_3_ treatment led to higher rice processing quality at the high altitude. The chalky grain rate and chalkiness degree at different altitudes increased with the increase in the proportion of postponed N fertilizer application.

**Table 5:** Effects of N management on rice appearance and processing quality under different altitude locations (2023-2024).

Year	Altitude	Variety	N management	Brown rice rate (%)	Milled rice rate (%)	Head rice rate (%)	Chalky grain rate (%)		Chalkiness ratio (%)	Length-width ratio
2023	Low altitude	MXZ 2	N_0_	75.25f	66.55e	59.00d	7.53d		8.93a	3.17a
			N_1_	75.51f	67.78d	59.32d	3.57f		5.28d	3.20a
			N_2_	77.53d	70.13a	61.60c	3.87ef		5.57cd	3.22a
			N_3_	76.29e	69.37bc	59.39d	4.67e		7.58b	3.18a
			**Average**	**76.15**	**68.46**	**59.83**	**4.91**		**6.84**	**3.19**
		YJ 39	N_0_	81.68c	66.94e	61.94bc	27.43a		9.43a	2.11c
			N_1_	82.62b	68.25d	62.59ab	23.84c		5.61cd	2.15bc
			N_2_	83.76a	70.03ab	63.05a	24.47bc		6.37c	2.23b
			N_3_	83.14b	69.01c	62.63ab	25.46b		7.96b	2.20bc
			**Average**	**82.80**	**68.56**	**62.55**	**25.30**		**7.34**	**2.17**
	High altitude	MXZ 2	N_0_	77.26f	67.63e	63.93e	2.61e		4.12b	2.89b
			N_1_	77.58f	69.19d	64.83d	1.24f		3.81b	3.02a
			N_2_	78.23e	70.26c	66.13cd	1.79ef		3.92b	3.04a
			N_3_	79.64d	70.84c	66.91bc	2.06ef		4.03b	3.06a
			**Average**	**78.18**	**69.48**	**65.45**	**1.92**		**3.97**	**3.00**
		YJ 39	N_0_	83.33c	70.96c	65.76cd	13.68a		6.32a	2.10d
			N_1_	84.78b	71.98b	66.48bc	5.80d		3.82b	2.13cd
			N_2_	85.12ab	72.94a	67.54ab	7.08c		3.84b	2.17cd
			N_3_	85.51a	73.18a	68.77a	11.92b		3.94b	2.200c
			**Average**	**84.69**	**72.27**	**67.14**	**9.62**		**4.48**	**2.15**
		*F* value	A	395.45**	267.26**	275.46**	2284.56**		837.87**	45.41**
			V	4459.18**	99.27**	92.54**	5170.12**		22.89**	3519.57**
			N	76.90**	81.00**	50.27**	92.72**		132.05**	7.98**
			A×V	0.55	86.19**	21.11**	1056.42**		0.07	28.21**
			A×N	14.77**	3.96*	20.38**	5.41**		33.84**	1.30
			V×N	4.20*	1.73	3.30*	16.55**		8.45**	0.51
			A×V×N	3.13*	0.21	2.61	18.59**		7.74**	1.15
2024	Low altitude	MXZ 2	N0	75.28f	68.05d	58.02d	6.89e		9.89bc	3.03b
			N1	77.25e	68.21d	58.91c	4.52g		7.52de	3.20a
			N2	78.65d	71.33b	61.17ab	5.27fg		9.07c	3.23a
			N3	78.38d	70.87c	59.30c	5.48f		9.28c	3.20a
			**Average**	**77.39**	**69.62**	**59.35**	**5.54**		**8.94**	**3.17**
		YJ 39	N0	81.69c	70.59c	58.34d	34.45a		11.05a	2.00e
			N1	81.91bc	71.33b	59.35c	25.09d		7.09e	2.10d
			N2	82.63a	72.95a	61.55a	26.93c		8.03d	2.20c
			N3	82.29ab	72.69a	60.57b	28.21b		10.32ab	2.17c
			**Average**	**82.13**	**71.89**	**59.95**	**28.67**		**9.12**	**2.12**
	High altitude	MXZ 2	N0	76.78e	71.17f	67.21e	2.63d		2.54c	2.93a
			N1	78.67d	72.08e	67.79de	1.41f		2.18e	2.93a
			N2	78.94d	72.57de	68.45c	2.14e		2.34de	2.97a
			N3	79.39d	73.18d	69.40b	2.37de		2.42cd	3.00a
			**Average**	**78.45**	**72.25**	**68.21**	**2.14**		**2.37**	**2.96**
		YJ 39	N0	85.07c	75.57c	68.16cd	6.37a		3.23a	1.97c
			N1	85.46c	75.83c	69.14b	3.40c		2.43cd	2.07b
			N2	86.47b	77.12b	70.96a	4.68b		2.99b	2.10b
			N3	87.31a	78.36a	71.52a	6.07a		3.12ab	2.13b
			**Average**	**86.08**	**76.72**	**69.95**	**5.13**		**2.94**	**2.07**
		F value	A	424.33**	1553.04**	9933.83**	10578.05**		14.01**	106.78**
			V	2594.76**	1269.04**	168.85**	9941.56**		66.73**	6032.11**
			N	65.05**	159.84**	162.00**	163.42**		3.780	28.56**
			A×V	141.83**	134.39**	28.01**	5908.28**		33.22**	40.11**
			A×N	2.57	13.28**	21.36**	47.34**		8.98**	3.96**
			V×N	10.31**	1.06	9.11**	52.56**		6.25**	1.00
			A×V×N	4.64**	10.25**	3.46*	22.23**		14.01**	1.90

MXZ 2, Meixiangzhan 2; YJ 39, Yunjing 39; N_0_ represents a blank control with no N fertilizer application, N_1_, N_2_, and N_3_ represent three N management strategies with a total N application rate of 150 kg ha^-1^ and basal:tiller:panicle fertilizer ratios of 5:3:2, 3:3:4, and 3:1:6, respectively. Different letters after the same column data indicate significant differences at the 5% level between treatments in the same location. A, Altitude; V ,variety; N, N management; A×V, altitude and variety management interaction; A×N, altitude and N management interaction; V×N, variety and N management interaction; A×V×N, altitude, variety and N management interaction. *, ** indicate significant differences at the 0.05 and 0.01 levels, respectively.

Bold values represent the average values of the same indicator for the same variety under four nitrogen fertilizer treatments. The lowercase letters in the table are used for statistical significance marking. Different letters indicate significant differences among treatments at the same ecological site at the 5% level, while the same letter indicates no significant difference.

**Figure 3:**
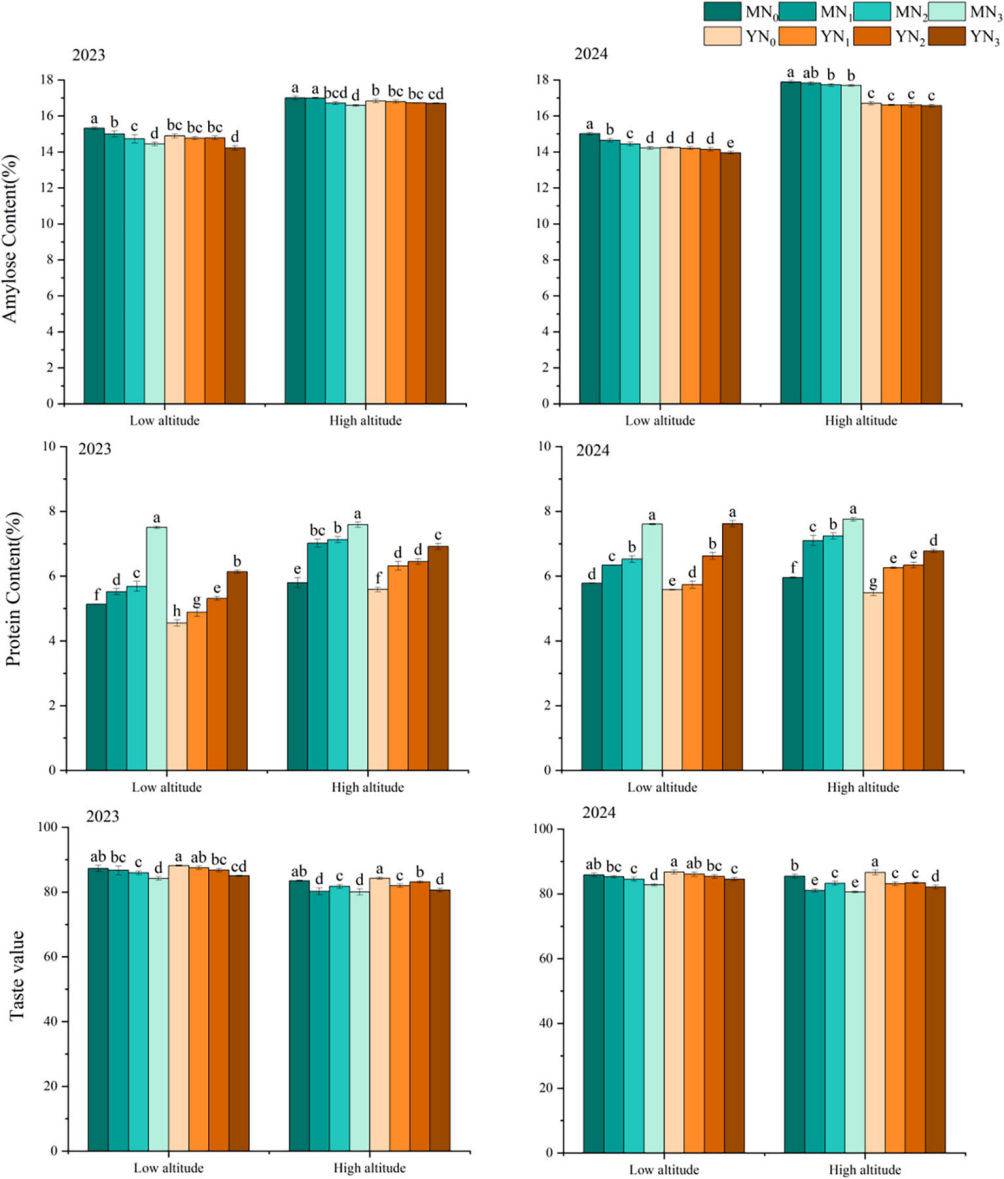
Effects of N management on rice nutritional and taste quality at different altitude locations (2023-2024). MN_0_, MN_1_, MN_2_, and MN_3_ represent the treatment combinations of the conventional *indica* rice variety Meixiangzhan 2 under four N treatments (N_0_: no N application; N_1_: basal: tillering: panicle fertilizer ratios of 5:3:2; N_2_: basal: tillering: panicle fertilizer ratios of 3:3:4; N_3_: basal: tillering: panicle fertilizer ratios of 3:1:6), respectively. YN_0_, YN_1_, YN_2_, and YN_3_ represent the treatment combinations of the conventional *japonica* rice variety Yunjing 39 under four N treatments (N_0_: no N application; N_1_: basal: tillering: panicle fertilizer ratios of 5:3:2; N_2_: basal: tillering: panicle fertilizer ratios of 3:3:4; N_3_: basal: tillering: panicle fertilizer ratios of 3:1:6), respectively. The error bars in the Figure represent the standard deviation. Different lowercase letters indicate significant differences between different crop rotation systems at the 0.05 level.

**Figure 3** demonstrates the significant effect of altitude on amylose content, protein content, and taste value, with consistent results across the two years. Compared with the low-altitude region, the amylose content and protein content at the high altitude increased significantly by 13.71% to 19.82% and 2.13% to 18.03%, respectively, while the taste value decreased by 2.29% to 5.22%. Compared with Meixiangzhan 2, the amylose content and protein content of Yunjing 39 were lower at different altitudes, and the taste value was better. The amylose content of each variety gradually decreased with the increase of the proportion of postponed N fertilizer application, while the protein content showed an increasing trend.

### 3.4 Characteristics and utilization of temperature and light resources across growth stages

**Table 6** shows that compared with the low altitude site, the average daily temperature, diurnal temperature range, and daily average sunshine hours at the high altitude increased by 0.48 to 2.28°C, 1.61 to 2.31°C, and 3.47 to 3.51 h, respectively, from sowing to jointing. The average daily temperature, diurnal temperature range, and daily average sunshine hours from jointing to heading decreased by 1.84 to 1.86°C, 0.82 to 1.19°C, and 0.31 to 0.34 h, respectively. From heading to maturity, rice in the low-altitude region experienced higher average daily temperature, while rice in the high-altitude region experienced higher diurnal temperature range and daily average sunshine hours. In the main growth period, the two varieties in the same ecological site experienced no significant difference in average daily temperature, diurnal temperature range, and daily average sunshine hours across N fertilizer treatments and growth stages.

**Table 6:** Average daily temperature, diurnal temperature range, sunshine hours at the main growth stages of rice in different altitude locations (2023-2024).

Year	Altitude	Variety	N management	Sowing-jointing stage			Jointing-heading stage			Heading-maturity stage		
				ADT (°C)	DTR (°C)	SH (h)	ADT (°C)	DTR (°C)	SH (h)	ADT (°C)	DTR (°C)	SH (h)
2023	Low altitude	MXZ 2	N_0_	20.81	10.07	3.45	26.19	10.90	5.24	26.44	9.29	5.01
			N_1_	20.83	10.12	3.55	26.26	10.80	5.14	26.54	9.41	5.10
			N_2_	20.82	10.11	3.58	26.27	10.78	5.19	26.42	9.47	5.12
			N_3_	20.86	10.13	3.50	26.29	10.78	5.13	26.62	9.20	4.90
		YJ 39	N_0_	20.72	10.11	3.41	25.91	10.75	4.78	26.36	9.30	5.10
			N_1_	20.78	10.08	3.47	26.04	10.87	4.90	26.35	9.56	5.00
			N_2_	20.75	10.08	3.53	26.07	10.90	5.03	26.24	9.50	5.22
			N_3_	20.73	10.06	3.46	26.06	10.91	5.03	26.55	9.20	5.02
			**Average**	**20.79**	**10.10**	**3.49**	**26.14**	**10.84**	**5.06**	**26.44**	**9.37**	**5.06**
	High altitude	MXZ 2	N_0_	23.05	12.39	6.95	24.27	9.66	4.72	22.17	8.56	6.15
			N_1_	23.12	12.43	7.02	24.13	9.44	4.47	22.54	8.36	5.90
			N_2_	23.12	12.45	7.10	24.14	9.44	4.42	22.24	8.49	6.00
			N_3_	23.09	12.43	7.06	24.14	9.43	4.42	22.43	8.35	5.87
		YJ 39	N_0_	23.04	12.37	6.90	24.47	9.98	5.18	22.11	8.53	6.11
			N_1_	23.04	12.39	6.95	24.43	9.75	4.88	22.47	8.43	5.96
			N_2_	23.05	12.40	6.96	24.42	9.73	4.86	22.10	8.60	6.26
			N_3_	23.05	12.39	7.00	24.43	9.73	4.83	22.31	8.46	5.99
			**Average**	**23.07**	**12.41**	**7.00**	**24.30**	**9.65**	**4.72**	**22.30**	**8.47**	**6.03**
2024	Low altitude	MXZ 2	N_0_	21.20	9.53	3.55	25.01	7.81	2.43	26.65	9.08	4.08
			N_1_	21.26	9.55	3.57	25.00	7.73	2.38	26.45	9.22	4.00
			N_2_	21.28	9.54	3.57	25.03	7.56	2.31	26.40	9.30	4.30
			N_3_	21.25	9.54	3.55	25.02	7.55	2.30	26.75	9.02	3.93
		YJ 39	N_0_	21.07	9.59	3.53	24.84	8.02	2.54	26.73	9.36	4.02
			N_1_	21.11	9.60	3.51	24.83	8.06	2.58	26.74	9.49	3.95
			N_2_	21.14	9.58	3.52	24.96	8.11	2.69	26.70	9.32	4.10
			N_3_	21.11	9.55	3.52	24.96	8.11	2.69	26.94	9.22	3.80
			**Average**	**21.18**	**9.56**	**3.54**	**24.96**	**7.87**	**2.49**	**26.67**	**9.25**	**4.02**
	High altitude	MXZ 2	N_0_	21.66	11.17	6.81	23.05	6.91	2.04	21.65	8.62	6.14
			N_1_	21.61	11.06	6.86	23.33	7.34	2.63	21.81	8.48	6.00
			N_2_	21.60	11.07	7.00	23.33	7.34	2.65	21.76	8.51	6.11
			N_3_	21.60	11.06	6.93	23.34	7.41	2.63	21.85	8.45	6.16
		YJ 39	N_0_	21.67	11.26	7.05	22.91	6.87	1.83	21.85	8.82	6.39
			N_1_	21.70	11.23	7.10	22.93	6.81	1.88	22.07	8.73	6.27
			N_2_	21.72	11.24	7.18	22.94	6.83	1.90	21.73	8.89	6.47
			N_3_	21.70	11.24	7.13	22.94	6.85	1.87	22.06	8.73	6.27
			**Average**	**21.66**	**11.17**	**7.01**	**23.10**	**7.05**	**2.18**	**21.85**	**8.65**	**6.23**

MXZ 2, Meixiangzhan 2; YJ 39, Yunjing 39; N_0_ represents a blank control with no N fertilizer application, N_1_, N_2_, and N_3_ represent three N management strategies with a total N application rate of 150 kg ha^-1^ and basal:tiller:panicle fertilizer ratios of 5:3:2, 3:3:4, and 3:1:6, respectively. ADT, average daily temperature; DTR, diurnal temperature range; SH, average daily sunshine hours.

Bold values represent the average values of the same indicator for the same variety under four nitrogen fertilizer treatments. The lowercase letters in the table are used for statistical significance marking. Different letters indicate significant differences among treatments at the same ecological site at the 5% level, while the same letter indicates no significant difference.

**Figure 4:**
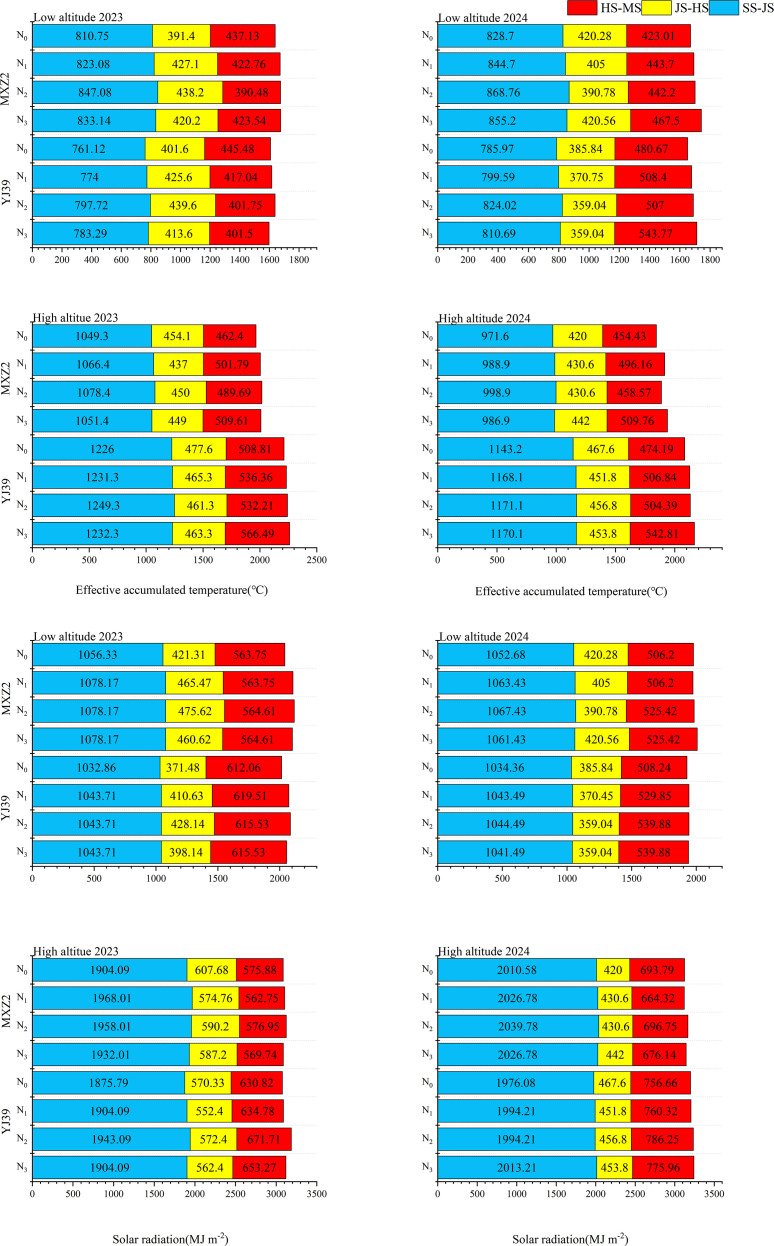
Effective accumulated temperature and solar radiation of main growth stages of rice in different altitude locations. MXZ 2: Meixiangzhan 2; YJ 39: Yunjing 39; N_0_ represents a blank control with no N fertilizer application, N_1_, N_2_, and N_3_ represent three N management strategies with a total N application rate of 150 kg ha^-1^ and basal:tiller:panicle fertilizer ratios of 5:3:2, 3:3:4, and 3:1:6, respectively. SS-JS: sowing to the jointing stage; JS-HS: jointing to heading stage; HS-MS: heading to maturity stage.

According to **Figure 4**, the effective accumulation of temperature and solar radiation in the whole rice growth period, from sowing to jointing, and from jointing to heading in the high-altitude region are significantly higher than that in the low-altitude region. However, the effective accumulated temperature from heading to maturity was high in the low-altitude region, while the advantage of solar radiation at the high altitude was obvious. Over the two years, the test results showed a consistent trend. From the perspective of N fertilizer management, since each N application treatment covered more days from sowing to jointing than the N_0_ treatment, the effective accumulated temperature and solar radiation before jointing were higher in the whole growth period at different altitudes.

**Figure 5:**
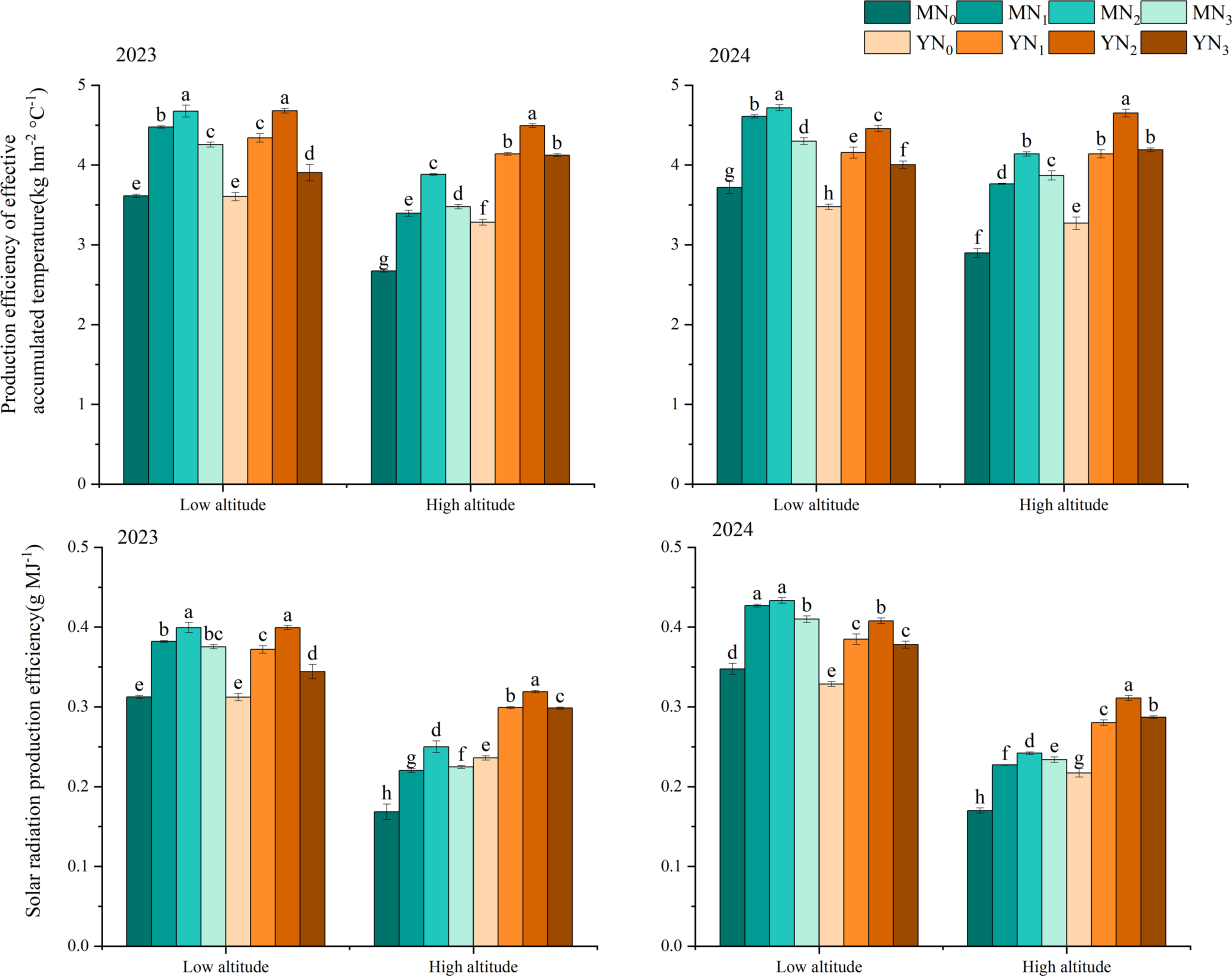
Effects of N management on temperature and light use efficiency of rice in different altitude location (2023-2024). MN_0_, MN_1_, MN_2_, and MN_3_ represent the treatment combinations of the conventional *indica* rice variety Meixiangzhan 2 under four N treatments (N_0_: no N application; N_1_: basal: tillering: panicle fertilizer ratios of 5:3:2; N_2_: basal: tillering: panicle fertilizer ratios of 3:3:4; N_3_: basal: tillering: panicle fertilizer ratios of 3:1:6), respectively. YN_0_, YN_1_, YN_2_, and YN_3_ represent the treatment combinations of the conventional *japonica* rice variety Yunjing 39 under four N treatments (N_0_: no N application; N_1_: basal: tillering: panicle fertilizer ratios of 5:3:2; N_2_: basal: tillering: panicle fertilizer ratios of 3:3:4; N_3_: basal: tillering: panicle fertilizer ratios of 3:1:6), respectively. The error bars in the Figure represent the standard deviation. Different lowercase letters indicate significant differences between different crop rotation systems at the 0.05 level.

**Figure 5** shows that altitude has a significant impact on the production efficiency of effective accumulated temperature and solar radiation production efficiency. Due to the delayed growth period at the high altitude (**Table 2**), the production efficiency of effective accumulated temperature and solar radiation production efficiency at the low altitude exceeded those at the high altitude. The production efficiency of effective accumulated temperature and solar radiation production efficiency of each variety differed across altitudes. The production efficiency of effective accumulated temperature and solar radiation production efficiency of Meixiangzhan 2 in the low-altitude region were higher than those of Yunjing 39, while those in the high-altitude region were lower than those of Yunjing 39 to varying degrees. At different altitudes, the production efficiency of effective accumulated temperature and solar radiation production efficiency of each variety increased first and then decreased with the increase in the proportion of postponed N fertilizer application, consistent with the yield trend (**Table 3**).

### 3.5 The relationship of temperature and light characteristics with rice yield and quality in the main growth stages at different altitudes

**Figure 6:**
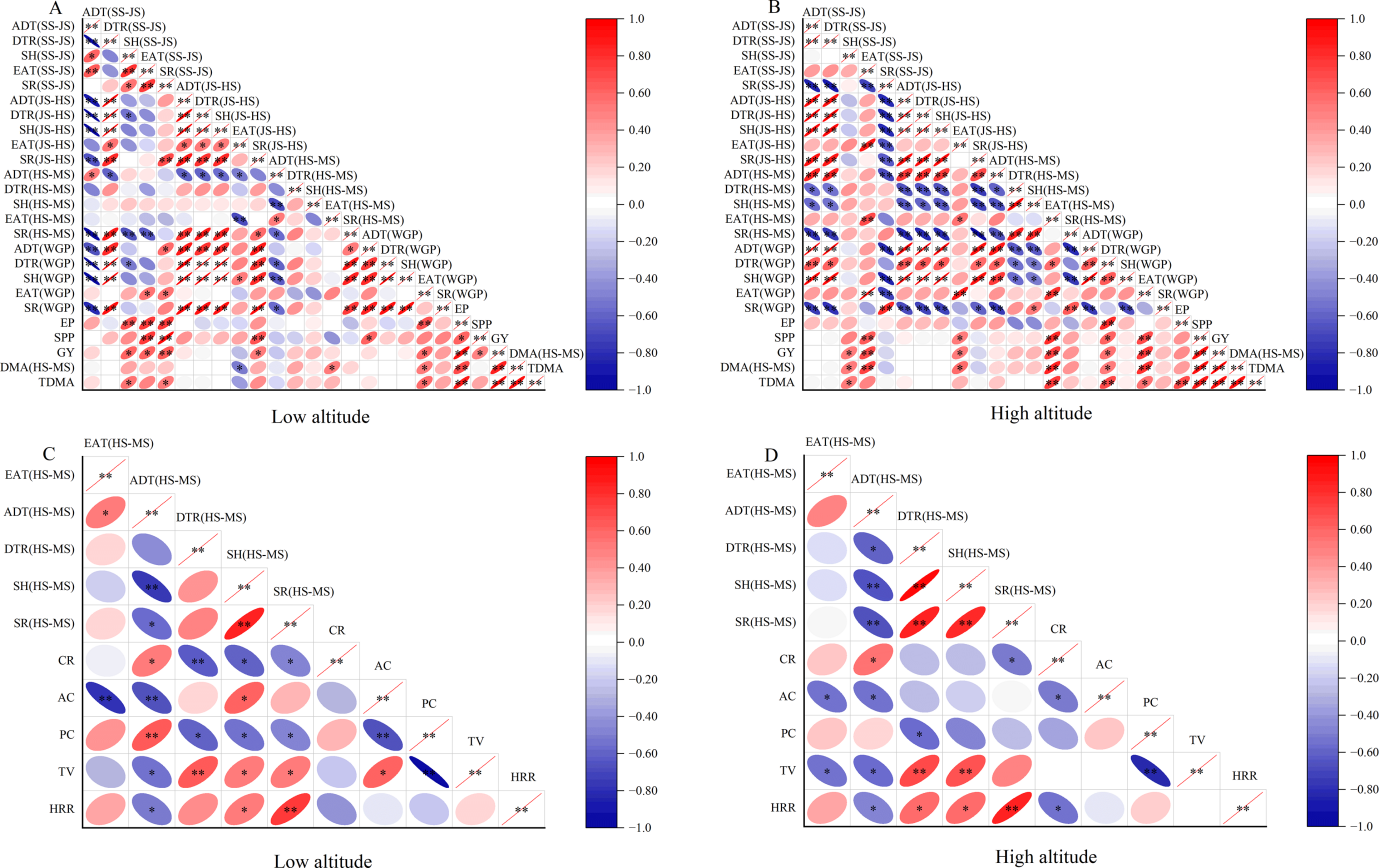
Correlation analysis of yield and quality formation with temperature and light characteristics at different altitudes. EP, effective panicles; GNPP, grain number per panicle; GY, grain yield; DMA, dry matter accumulation; TDMA, total dry matter accumulation; DMTR, dry matter transport rate of stem-sheath and leaf; HRR, head rice rate; CR, chalkiness ratio; AC, amylose content; PC, protein content; TV, taste value; SS-JS, sowing to jointing stage; JS-HS, jointing to heading stage; HS-MS, heading to maturity stage; WGP, whole growth period; ADT, average daily temperature; EAT, effective accumulated temperature; DTR, diurnal temperature range; SH, average daily sunshine hours; SR, solar radiation. *, ** indicate significant differences at the 0.05 and 0.01 levels, respectively.

The correlation analysis (**Figure 6**) showed that the yield at the low altitude was significantly positively correlated with the average daily sunshine hours, effective accumulated temperature, and solar radiation from sowing to jointing. Specifically, the yield was significantly positively correlated with solar radiation from jointing to heading stage and the effective accumulated temperature during the whole growth period. The yield in the high-altitude region was significantly positively correlated with the average daily sunshine hours and effective accumulated temperature from sowing to jointing. Specifically, the yield was significantly positively correlated with the effective accumulated temperature from jointing to heading, the effective accumulated temperature from heading to maturity, and the effective accumulated temperature during the whole growth period. Comprehensively, the average daily sunshine hours, effective accumulated temperature, and effective accumulated temperature during the whole growth period and from sowing to jointing can be leveraged as meteorological diagnostic indicators to evaluate the synergistic improvement of rice yield and quality across altitudes.

In the low-altitude region, the chalkiness of rice was significantly positively correlated with average daily temperature and significantly negatively correlated with diurnal temperature range, daily average sunshine hours, and solar radiation. The amylose content was significantly negatively correlated with effective accumulated temperature and average daily temperature, but was significantly positively correlated with daily average sunshine hours. The protein content was significantly positively correlated with the effective accumulated temperature but significantly negatively correlated with the diurnal temperature range, daily average sunshine hours, and solar radiation. The taste value was significantly negatively correlated with average daily temperature and was significantly positively correlated with diurnal temperature range, daily average sunshine hours, and solar radiation. The head rice rate was significantly negatively correlated with average daily temperature and was significantly positively correlated with daily average sunshine hours and solar radiation. In the high altitude, the chalkiness degree was significantly positively correlated with average daily temperature and significantly negatively correlated with solar radiation. The amylose content was significantly negatively correlated with effective accumulated temperature and average daily temperature. The protein content was significantly negatively correlated with the diurnal temperature range. The taste value was significantly negatively correlated with effective accumulated temperature and average daily temperature, but was significantly positively correlated with diurnal temperature range and daily average sunshine hours. The head rice rate was significantly negatively correlated with average daily temperature and significantly positively correlated with diurnal temperature range, daily average sunshine hours, and solar radiation. These results showed that the higher diurnal temperature range, daily average sunshine hours, and solar radiation from heading to maturity could improve the processing and eating quality of rice, but could lead to decreased nutritional quality. The higher effective accumulated temperature and average daily temperature from heading to maturity could reduce the appearance, processing, and eating quality of rice.

### 3.6 Principal component analysis and evaluation of rice yield and quality across altitudes and N fertilizer managements

**Table 7** shows that the cumulative contribution rate of the two principal components at the low altitude is 87.54%, and that at the high altitude was 91.59%. The contribution rate of principal component 1 at the low altitude was 67.87%, which mainly reflected the information of indicators like grain yield, head rice rate, chalkiness, dry matter accumulation from heading to maturity, total dry matter accumulation, production efficiency of effective accumulated temperature, and solar radiation production efficiency. The contribution rate of principal component 2 was 19.67%, which mainly reflected the information of indicators such as taste value and protein content. The contribution rate of principal component 1 was 66.17% at the high altitude, which mainly reflected the information of indicators such as grain yield, head rice rate, dry matter accumulation from heading to maturity, total dry matter accumulation, production efficiency of effective accumulated temperature, and solar radiation production efficiency. The contribution rate of principal component 2 was 25.42%, which mainly reflected the information of chalkiness, taste value, and protein content.

**Table 7:** Eigen values and contribution ratio of principle component.

	Principle component	Low altitude		High altitude	
		The first principal component PC1	The second principal component PC2	The first principal component PC1	The second principal component PC2
Eigen value		6.108	1.770	5.995	2.288
Contribution ratio		67.872	19.665	66.172	25.419
Cumulative contribution ratio (%)		67.872	87.537	66.172	91.590
Eigen vector	Grain yield	0.980	0.019	0.949	0.289
	Head rice rate	0.464	0.293	0.895	0.202
	Chalkiness	-0.820	-0.440	-0.352	0.726
	Protein content	0.484	-0.864	0.442	-0.832
	Taste value	-0.540	0.838	-0.504	0.807
	Dry matter accumulation from heading to maturity	0.933	0.121	0.949	0.277
	Total dry matter accumulation	0.988	0.082	0.996	-0.075
	Production efficiency of effective accumulated temperature	0.963	0.142	0.967	0.082
	Solar radiation production efficiency	0.979	0.025	0.943	0.306

According to **Table 8**, the weights of the two comprehensive indicators at different altitudes were calculated as 0.775 and 0.225 and 0.722 and 0.278, respectively. According to the comprehensive evaluation of indicator weight and membership function value, Yunjing 39 under the N_2_ treatment achieved the highest score at the high altitude, while Meixiangzhan 2 under the N_2_ treatment achieved the highest score at the low altitude. The comprehensive scores of the four N fertilizer management modes on rice yield and quality ranked as N_2_>N_3_>N_1_>N_0_ at the high altitude, but N_2_>N_1_>N_3_>N_0_ at the low altitude.

**Table 8:** Comprehensive evaluation of different treatments on rice productivity and quality.

Treatment			Integrated index value		Subordinate function value		Comprehensive evaluation value	Rank
			PC1	PC2	μ(X1)	μ(X2)	D-value	
Low altitude	MXZ 2	N_0_	-1.391	-0.133	0.114	0.566	0.216	7
		N_1_	0.635	0.401	0.886	0.735	0.852	3
		N_2_	0.934	0.205	1.000	0.673	0.927	1
		N_3_	0.439	-1.917	0.812	0.000	0.629	5
	YJ 39	N_0_	-1.690	0.439	0.000	0.748	0.168	8
		N_1_	0.287	1.235	0.753	1.000	0.809	4
		N_2_	0.811	0.710	0.953	0.834	0.926	2
		N_3_	-0.025	-0.940	0.634	0.310	0.562	6
Weight					0.775	0.225		
High altitude	MXZ 2	N_0_	-1.738	-0.088	0.000	0.388	0.108	8
		N_1_	-0.319	-1.187	0.494	0.013	0.360	7
		N_2_	0.094	-0.560	0.637	0.227	0.523	4
		N_3_	0.123	-1.227	0.647	0.000	0.468	5
	YJ 39	N_0_	-0.997	1.709	0.258	1.000	0.464	6
		N_1_	0.604	0.290	0.815	0.517	0.732	3
		N_2_	1.137	0.845	1.000	0.706	0.918	1
		N_3_	1.097	0.218	0.986	0.492	0.849	2
Weight					0.722	0.278		

PC1, The first principal component; PC2, The second principal component; μ(X1), Membership function value of the first principal component; μ(X2), Membership function value of the second principal component.

## 4 Discussion

### 4.1 Analysis of rice yield across altitudes

The ecological environments at different altitudes significantly affected rice yield (Chen et al., 2023b). Comparative studies across ecological sites at different altitudes in the Sichuan rice-growing region have indicated that sufficient solar radiation at high altitudes promotes the synergistic increase of effective panicle number and grain number per panicle, collectively ensuring high rice yields in these areas (Zhong et al., 2022). However, other researchers, by analyzing the yield changes of different rice varieties along altitudinal gradients, have drawn the opposite conclusion: in extremely high-altitude areas of the southwestern rice region, yield declines with increasing altitude, a phenomenon attributed to the concurrent reduction in both effective panicle number and grain number per panicle (Zheng et al., 2020a). In the present study, yield at the high-altitude ecological site increased by 4.10%-4.12%. The underlying mechanism lies in the non-linear relationship between altitude and yield in the southwestern rice region: within an optimal altitude window, lower temperatures and higher cumulative solar radiation after heading prolonged the rice growth period (**Table 2**) and optimized the source-sink relationship. Sufficient daytime solar radiation enhanced photosynthetic carbon assimilation, whereas cool nighttime temperatures reduced respiratory consumption. These physiological changes promoted dry matter accumulation and its translocation from rice stems and leaves (Peng et al., 2004; Tu et al., 2022), thereby increasing the effective panicle and seed setting rate of both varieties (**Table 3**). This is the main source of the yield difference between the two ecological sites. This was the main source of yield differences between the two ecological points. In addition, this study identified the effective panicle number and thousand kernel weight, respectively, as the main factors affecting the yield difference between conventional indica rice and conventional japonica rice in the low-altitude and high-altitude regions. Low temperatures during grain filling can affect spikelet fertility, hinder the normal development of pollen, and then lead to yield reduction (Giorno et al., 2013). In this study, the grain number per panicle of the two varieties decreased to varying degrees as the altitude increased, which further reinforced the conclusions of Giorno et al. This may also be related to the fact that the increased average daily temperature from heading to maturity synergistically increased the grain number per panicle.

Previous studies have shown that appropriate N management improves rice growth, dry matter accumulation, dry matter translocation rate, and dry matter contribution rate, thereby promoting rice yield (He et al., 2024). Heavy basal and tiller fertilizer application can lead to excessive development of leaf area, resulting in a deteriorated light environment in the population and, in turn, declined photosynthetic characteristics (Chen et al., 2018). In contrast, an excessive proportion of panicle fertilizer, although it increases the panicle rate of tillers, leads to insufficient peak seedling numbers and a subsequent reduction in effective panicles at later stages. Moreover, it suppresses both the seed setting rate and thousand kernel weight, ultimately resulting in decreased rice yield (Wu et al., 2020). In this study, the yield was the highest under the N_2_ treatment, as it facilitated higher total dry matter accumulation, total spikelet number, thousand kernel weight, and the stem and leaf material translocation efficiency.

### 4.2 Analysis of rice quality under different ecological conditions across altitudes

Rice quality covers the processing, appearance, nutritional, cooking, and eating dimensions (Liu et al., 2014), which are essentially determined by genetic and environmental factors (Gong et al., 2023). It is generally believed that the filling stage is key to rice quality (Lu et al., 2024). Studies conducted at three ecological sites in the karst landscapes of the southwestern rice region have demonstrated that low temperatures at high altitudes significantly reduce rice chalkiness and improve processing quality (Xia et al., 2024). This study found that, compared with the lower altitude, the higher altitude increased the brown rice rate, milled rice rate, and head rice rate of conventional indica and conventional japonica rice to varying degrees, whereas the chalky grain rate and chalkiness decreased to varying degrees. With the increase in altitude, rice processing and appearance quality improved. Correlation analysis showed that the average daily temperature from heading to maturity was inversely proportional to the head rice rate and positively proportional to chalkiness. This may be explained by the higher temperature and lower solar radiation after heading in the low-altitude region, which accelerated the grain filling rate and shortened the grain filling time. The resultant starch granule formation and arrangement alterations led to a significant increase in chalkiness degree and chalky grain rate and a less compact starch structure that rendered the rice grain prone to breaking during processing (Huo et al., 2012). N fertilizer management is one of the primary cultivation measures explaining the differences in rice appearance and processing quality. Panicle N fertilizer is beneficial for rice processing quality, but increases the chalky grain rate and chalkiness and reduces the appearance quality (Wu et al., 2022b). This study showed that appropriate proportions of N fertilizer in different ecological sites could improve the processing quality of the two varieties, while the appearance quality showed different degrees of deterioration. These results further complement and enrich previous research findings.

The amylose content and protein content are important indicators of the nutritional quality of rice, which also significantly affect the eating quality. It is generally believed that the high protein content and the high degree of short-range ordered structure of starch inhibit starch pasting and reduce the eating quality (Ma et al., 2024). Some researchers, based on field experiments in the Yangtze River Basin, have concluded that high temperatures reduce the cooking and eating quality of rice (Dou et al., 2017). In this research, the amylose and protein content in the high-altitude region exceeded that in the low-altitude region, while the taste value was lower than that in the low-altitude region, inconsistent with the conclusion of Dou et al. Following the initial experimental study, excessively low or high temperatures during maturation may reduce the taste of cooked rice (Hu et al., 2020), the amylose content was negatively correlated with the temperature (average temperature, temperature difference, effective accumulated temperature, maximum temperature, and minimum temperature) from heading to maturity (Xia et al., 2024). In this study, the amylose content was significantly negatively correlated with the average daily temperature (**Figure 6**). In high-altitude regions, the average daily temperature remains relatively lower during the grain-filling stage from heading to physiological maturity. Moderately low temperatures significantly enhance the activity of granule-bound starch synthase (GBSS), a key enzyme responsible for amylose biosynthesis. This elevated GBSS activity increases the amylose proportion in the amylose–amylopectin ratio (Ahmed et al., 2015), which restricts the swelling of starch granules during cooking. Consequently, rice grain exhibits inferior eating quality at high altitudes relative to that at low-altitude sites. The differences in rice nutritional and cooking quality are also closely related to N fertilizer management, moderate panicle N fertilizer application would reduce the amylose content and increase the protein content (Xiong et al., 2024). In this study, the amylose and protein content across varieties and altitudes were the highest under the N_1_ and N_3_ treatments, respectively, which further complemented the previous research results. The results also showed that the amylose content gradually decreased while the protein content increased with the increase in the proportion of postponed N fertilizer. Therefore, an effective way to improve rice quality is to coordinate the proportion of N application at different rice growth stages based on different altitudes.

### 4.3 Efficient utilization of temperature and light resources for rice yield and quality improvements and efficiency enhancement at different altitudes

The obvious three-dimensional climate conditions in the rice planting regions across Southwest China lead to quite different rice yield and quality at different altitudes. Exploring the relationship between rice yield and quality and the temperature and light characteristics at different altitudes is crucial for improving regional rice yield and quality. Studies have shown that the effective accumulated temperature after rice heading is significantly positively correlated with both the seed setting rate and yield. Short sunshine hours, insufficient accumulated temperature, and low diurnal temperature range reduced the thousand kernel weight and seed setting rate, resulting in decreased yield (Feng et al., 2022; Zheng et al., 2020b). Moreover, Reduced solar radiation during the rice growing season is a key ecological constraint to improving rice grain yield. Compared with rice cultivated at high-altitude sites with favorable light conditions, rice grown at low-altitude, low-light sites develops a significantly lower effective panicles and total spikelets, which is the primary driver of its lower grain yield (Jiang et al., 2025). In this study, rice yield was significantly positively correlated with the effective accumulated temperature of the entire growth period, indicating it as a factor that could synergistically increase rice yield, which further supplemented the previous research conclusions. It has been reported that higher average temperature at the heading stage thickened the rice bran layer, which in turn hindered the processing performance (Ezin et al., 2022). Meanwhile, high temperatures can induce accelerated grain filling rates and insufficient grain filling (Yao et al., 2020). In terms of appearance quality, extensive research has shown that the appearance quality of rice is significantly linked to the average temperature under different ecological conditions. Chalkiness is caused by insufficient grain filling and is positively correlated with temperature (Cheng et al., 2019; Yao et al., 2020). Light intensity and daily sunshine duration are also key factors governing the appearance quality of rice grains. Insufficient solar irradiance markedly suppresses the photosynthetic efficiency of rice leaves, restricts photosynthate biosynthesis and accumulation, and disrupts assimilate partitioning to developing grains. Consequently, total starch and amylose concentrations in rice grains decrease concomitantly, while starch granules exhibit a looser, more disordered arrangement with expanded intergranular voids; these changes ultimately drive a significant elevation in chalky grain percentage and chalkiness degree (Noori et al., 2022). In terms of nutritional and eating quality, high temperatures during grain filling can alter the composition and crystal structure of starch, resulting in poor eating and cooking quality of early indica rice (Zhong et al., 2005). Insufficient incident solar irradiance induces a marked reduction in grain amylose content and RVA paste breakdown value, while driving a significant elevation in paste setback value and grain crude protein content; these coordinated changes in grain physicochemical properties ultimately drive the deterioration of rice grain cooking and eating quality (Dong et al., 2013). In this study, the increased diurnal temperature range, daily average sunshine hours, and solar radiation from heading to maturity were identified as the main factors allowing rice to utilize temperature and light resources efficiently at different altitudes to obtain higher quality. However, the mechanistic basis of how the interaction between variety and nitrogen management modulates altitude-driven differences in rice yield and quality remains incompletely understood. We will therefore pursue this line of research in greater depth in future studies by integrating multi-omics approaches, with the ultimate goal of elucidating the molecular mechanisms that positively regulate rice yield and grain quality.

## 5 Conclusions

Altitude significantly affected rice yield and quality. Compared with low-altitude rice-growing regions, high-altitude rice-growing regions extend the rice growth period while increasing the effective panicle number, seed setting rate, and the stem and leaf dry matter translocation rate and contribution rate, which were important reasons for rice yield increase. The processing, appearance, and nutritional quality of rice were improved at the high altitude, but the accumulation of amylose caused by the low average daily temperature from heading to maturity led to decreased eating quality and lower temperature and light production efficiency. Under the N application rate of 150 kg ha^−1^, the treatment with a basal to tiller to panicle fertilizer ratio of 3:3:4 proved optimal for yield, quality, and efficiency improvements at different altitudes. Varieties with sufficient effective panicles and more grain number per panicle should be selected for low-altitude rice-planting regions, and varieties with more grain number per panicle and high thousand kernel weight should be selected for high-altitude rice-planting areas. In particular, increasing the effective accumulated temperature over the entire rice growth period, the diurnal temperature range from heading to maturity, the average daily sunshine hours, and the solar radiation are the key ways to synergistically improve rice yield and quality in different rice-planting regions.

## Data Availability

The original contributions presented in the study are included in the article/supplementary material. Further inquiries can be directed to the corresponding author.
